# Polarity signaling balances epithelial contractility and mechanical resistance

**DOI:** 10.1038/s41598-023-33485-5

**Published:** 2023-05-12

**Authors:** Matthias Rübsam, Robin Püllen, Frederik Tellkamp, Alessandra Bianco, Marc Peskoller, Wilhelm Bloch, Kathleen J. Green, Rudolf Merkel, Bernd Hoffmann, Sara A. Wickström, Carien M. Niessen

**Affiliations:** 1 Department Cell Biology of the Skin, University Hospital Cologne, University of Cologne, Cologne, Germany; 2 Cologne Excellence Cluster for Stress Responses in Ageing-Associated Diseases (CECAD), University of Cologne, Cologne, Germany; 3 Center for Molecular Medicine Cologne (CMMC), University Hospital Cologne, University of Cologne, Cologne, Germany; 4 Forschungszentrum Jülich, Institute of Biological Information Processing, IBI-2: Mechanobiology, 52428 Jülich, Germany; 5 Institute for Genetics, University of Cologne, Cologne, Germany; 6 Department of Molecular and Cellular Sport Medicine, Institute of Cardiovascular Research and Sport Medicine, German Sport University of Cologne, Cologne, Germany; 7 Departments of Pathology and Dermatology, Feinberg School of Medicine, Northwestern University, Chicago, IL 60611 USA; 8 Max Planck Institute for Biology of Ageing, 50931 Cologne, Germany; 9 Stem Cells and Metabolism Research Program, Faculty of Medicine, University of Helsinki, 00290 Helsinki, Finland

**Keywords:** Cytoskeleton, Cell adhesion

## Abstract

Epithelia maintain a functional barrier during tissue turnover while facing varying mechanical stress. This maintenance requires both dynamic cell rearrangements driven by actomyosin-linked intercellular adherens junctions and ability to adapt to and resist extrinsic mechanical forces enabled by keratin filament-linked desmosomes. How these two systems crosstalk to coordinate cellular movement and mechanical resilience is not known. Here we show that in stratifying epithelia the polarity protein aPKCλ controls the reorganization from stress fibers to cortical actomyosin during differentiation and upward movement of cells. Without aPKC, stress fibers are retained resulting in increased contractile prestress. This aberrant stress is counterbalanced by reorganization and bundling of keratins, thereby increasing mechanical resilience. Inhibiting contractility in aPKCλ^−/−^ cells restores normal cortical keratin networks but also normalizes resilience. Consistently, increasing contractile stress is sufficient to induce keratin bundling and enhance resilience, mimicking aPKC loss. In conclusion, our data indicate that keratins sense the contractile stress state of stratified epithelia and balance increased contractility by mounting a protective response to maintain tissue integrity.

## Introduction

Vertebrate terrestrial life was facilitated by the evolution of the skin as a robust barrier towards the outside environment. The outermost barrier-forming layer of the skin, the epidermis, is a unique multilayered epithelium that prevents dehydration while protecting against a variety of environmental stresses such as irradiation, chemical irritants, microbes and mechanical stress. To ensure lifelong tissue functionality in the face of these stresses, the epidermis renews by balancing proliferation of basal stem/progenitor cells with a stepwise terminal differentiation program. During this program keratinocytes change shape while moving upwards through the epidermal layers resulting in shedding of the dead, cornified cells from the outer surface^1–3^. Importantly, these dynamic cellular rearrangements take place while preserving tissue integrity and epidermal barrier function.

The contractile actomyosin cytoskeletal network is a key determinant of cell shape and motility. Cellular actomyosin force dynamics are communicated to neighbors through interaction with the cadherin-based adherens junctions (AJ), thus enabling force transmission and cell rearrangements. As a consequence, loss of function of either myosin, regulators of actin dynamics, or adherens junction components in the mouse epidermis results in impaired differentiation and/or barrier dysfunction^4–6^.

Keratins build the intermediate filament cytoskeleton of keratinocytes. Their filaments exhibit high bending flexibility and through sliding and unfolding support large elongations. This flexibility together with the property of keratins to stiffen upon strain provides cells with high resistance to mechanical stress^7–9^. The keratin network connects with the intercellular adhesive desmosomes, thus distributing mechanical energy supracellularly to preserve tissue integrity and barrier function. The importance of keratins and desmosomal components for the mechanical stability of the skin is illustrated by loss of function mouse mutants, human mutations or autoantibodies that result in epidermal fragility and skin blistering^10–12^.

Whereas the regulation of AJ-actomyosin dynamics has been extensively studied^13,14^, much less is known about the regulation and dynamics of the desmosome-keratin adhesion system^15^. Crosstalk between AJs and desmosomes is essential to establish mature intercellular junctions. Whereas cadherin adhesive and cytoskeletal interactions at AJ are essential to initiate desmosome assembly^16,17^, mature AJ formation and junctional actomyosin reorganization requires desmoplakin, which links keratins to desmosomes^18^. In agreement, desmoplakin regulates epithelial cell tension through changes in actomyosin^19^. Moreover, loss of keratins results in fragmented AJ and desmosomes in keratinocytes and profoundly alters actomyosin organization^20^. However, how these two systems cooperate to balance intercellular dynamics and mechanical resilience of the tissue and whether this coordination operates at the junctional or cytoskeletal level is largely unknown.

Due to its stratified structure, the network of junctional and cytoskeletal cooperation in the epidermis is complex^4,21^. Basal keratinocytes anchor actin and keratin5/keratin14 (K5/K14) keratin filaments to the basement membrane through focal adhesion and hemidesmosomes, respectively, and connect to lateral and apical neighbors through AJ and desmosomes. In contrast, suprabasal cells only use these intercellular junctions to connect actin and K1/K10 networks to neighboring keratinocytes in all directions^4^. Consequently, basal to suprabasal movement is associated with substantial reorganization of the F-actin cytoskeleton and a change in keratin composition. Cultured keratinocyte monolayers mimic basal cells and form concentric F-actin rings associated with radial stress fibers connecting to early junctions, whereas this network is rearranged when cells move suprabasally into a dense cortical F-actin network^22,23^. How cell position-dependent actin re-organization is regulated, and whether the re-positioning itself or the associated actin remodeling regulates basal versus suprabasal keratin organization and resilience is unknown.

The atypical kinase C (aPKC) is an evolutionary conserved polarity protein that controls cortical actomyosin contractility and reorganization to promote the maturation of actin-linked junctions i.e. AJ and TJ^24–27^. Given the intimate connections between AJ-actin and desmosome-keratin assembly and organization, we asked whether mammalian aPKCs, aPKCζ and aPKCλ, also regulate mechanical resilience by changing desmosomes and/or keratin organization directly, or indirectly through cell-position dependent changes in actomyosin. Surprisingly, we find that loss of aPKCλ but not of aPKCζ increased mechanical resilience of stratified keratinocytes sheets but not monolayer basal sheets, likely as a result of suprabasal keratin reorganization but without any obvious changes in desmosome structure. This mechanical adaptation of keratins occurred in response to an increase in suprabasal contractility due to an inability to properly reorganize the actomyosin network suprabasally upon loss of aPKC. The data indicate that keratins can sense and respond to the state of actomyosin and that aPKCλ functions as a rheostat essential for basal differentiating keratinocytes to reorganize the actomyosin network to lower contractility during upward movement.

## Results

### aPKC regulates stratified epithelial mechanical resilience

**Figure 1:**
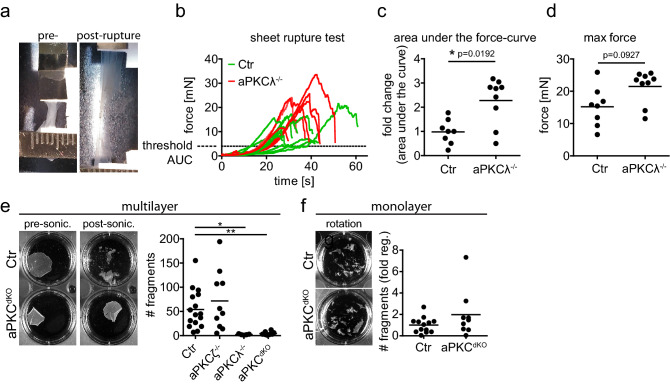
aPKC regulates mechanical resilience of stratified epithelial sheets. (**a**) Example of stratified keratinocyte sheet mounted in the tissue stretcher before (pre-) and after (post-) rupture. (**b**) Measured force over time while stretching with constant speed. (**c**) Quantification of the fold change of the area under the curve (AUC) from (**b**), normalized to the Ctr AUC mean value. With Mann–Whitney test for n = 8 (Ctr) and n = 9 (aPKCλ^−/−^). (**d**) Quantification of the maximum force that was reached during stretching, prior to sheet rupture. With Mann–Whitney test for n = 8 (Ctr) and n = 9 (aPKCλ^−/−^). Dots represent biological replicates. (**e**) Dispase assay with stratified keratinocyte multilayers (48 h Ca^2+^) after detachment upon dispase treatment. Images: Examples of sheets after end over end rotation, pre and post sonication. Graph: Quantification of sheet fragments upon sonication. *p < 0.05 with ANOVA followed by Dunnett’s multiple comparison test for n = 16 (Ctr), n = 5 (aPKCζ^−/−^), n = 10 (aPKCλ^−/−^), n = 10 (aPKC^dKO^). (**f**) Dispase assay with keratinocyte monolayers (6 h Ca^2+^) after detachment upon dispase treatment. Images: Examples of sheets after end over end rotation. Graph: Quantification of sheet fragments upon end over end rotation. n = 13 (Ctr), n = 9 (aPKC^dKO^).

To examine whether mammalian aPKCs regulate the mechanical resilience of multilayered stratified epithelia, e.g. the epidermis, we employed a micro-tissue stretcher^28^. In vitro stratified epidermal sheets (48 h differentiation in 1.8 mM high calcium medium) from primary control or aPKCλ^−/−^ keratinocytes were subjected to external pulling forces while measuring the resisting force of the sheet until rupture^28^ (Fig. 1a,b; Fig. 1 supplement a; Supplementary Video 1). Surprisingly, aPKCλ^−/−^ keratinocyte sheets resisted twice as much pulling force for rupture compared to control sheets (Fig. 1b–d).

To examine whether aPKC similarly regulates mechanical stability under shear stress, we performed keratinocyte dissociation assays in which keratinocyte sheets were exposed to different levels of shear force. Exposure to intermediate shear force (using end-over-end rotation) was insufficient to dissociate either control or aPKC deficient keratinocyte sheets. To increase shear stress sheets were then subjected to brief sonication, resulting in fragmentation of control but not aPKCλ^−/−^ sheets (Fig. 1e). Importantly, staining for desmoglein 3 (Dsg3) and DNA confirmed that sonication did not cause fragmentation through induction of cell lysis (Fig. 1 supplement b). Increased resistance to shear stress was specific for aPKCλ as aPKCζ^−/−^ sheets fragmented to a similar extent as controls, and combined loss of both aPKCs (aPKC^dKO^) did not change the phenotype of the aPKCλ single knockout (Fig. 1e).

We next asked whether aPKCλ also controls resilience of only basal keratinocytes. Keratinocyte monolayers that mimic only the basal epidermal layer (6 h differentiation in 1.8 mM high calcium medium), fragmented already during end-over-end rotation, also to a similar extend upon loss of aPKC (Fig. 1f). Taken together, these results show that keratinocyte sheet resilience increases with stratification and that aPKCλ regulates adaptation of mechanical stability in stratified epithelia either upon stratification or specifically in suprabasal layers.

### Increased resilience is independent of altered desmosomes

**Figure 2:**
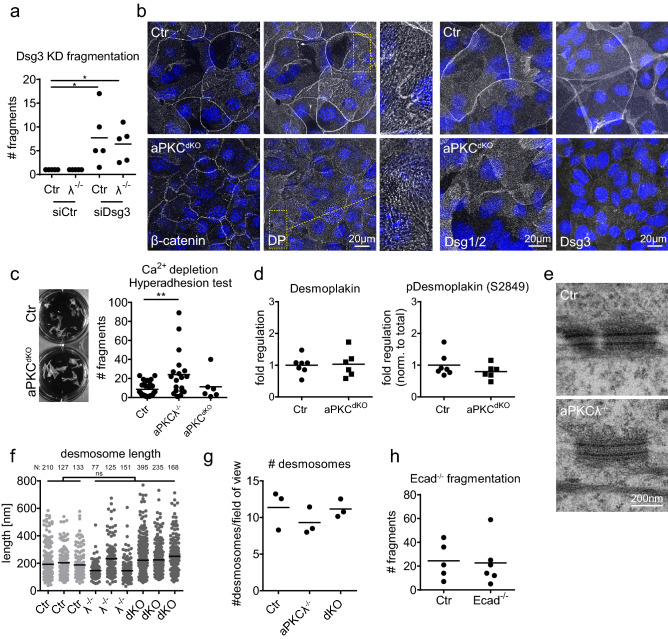
Increased resistance does not depend on alterations in desmosomes. (**a**) Dispase assay with stratified keratinocyte multilayers (48 h Ca^2+^) transfected with either ctr siRNA (siCtr) or siRNA against desmoglein3 (siDsg3). Quantification of sheet fragments upon end over end rotation: *P = 0.0116 with 1-way ANOVA, Tukey’s post test for Ctr/aPKCλ^−/−^, siCtr/siDsg3: *n* = 5. (**b**) Immunofluorescence staining for β-catenin, desmoplakin (DP), desmoglein1/2 (Dsg1/2) and desmoglein3 (Dsg3) in stratified keratinocyte multilayers (48 h Ca^2+^). Enlargements show punctate desmosomal intercellular junctions. Nuclei labeled with DAPI (blue). (**c**) Quantification of sheet fragments upon EGTA treatment and end over end rotation. **P = 0.009 with 1-way ANOVA, Tukey´s post test for Ctr: *n* = 26, aPKCλ^−/−^: *n* = 19, aPKC^dKO^: *n* = 6. (**d**) Western Blot quantification of total and phospho-desmoplakin levels (Ser2849) in lysates of stratified keratinocyte sheets (48 h Ca^2+^). (**e**) Transmission electron micrographs, cross section of stratified keratinocyte sheets 48 h in high Ca^2+^ showing comparable desmosome in Ctr and aPKCλ^−/−^ keratinocytes. Representative images from *n* = 3 (Ctr/aPKCλ^−/−^). (**f**, **g**) Quantification of desmosomal length (**f**) and quantity (**g**) from transmission electron micrographs as shown in (**e**) for Ctr/aPKCλ^−/−^/aPKC^dKO^: *n* = 3. Numbers of quantified desmosomes/biological replicate are indicated above the graph. (**h**) Dispase assay with stratified Ctr and E-cadherin^−/−^ keratinocyte multilayers (48 h Ca^2+^) after detachment upon dispase treatment. Graph: Quantification of sheet fragments upon sonication. n = 5 (Ctr), n = 6 (E-cadherin^−/−^).

Changes in stratified epithelial tissue stability have been linked to alterations in the mechanical properties of desmosomes and/or intermediate filaments^12^. We thus first asked whether desmosomal function was required for increased resilience and depleted one of the major desmosomal adhesion receptors, desmoglein-3 (Dsg3). Knockdown of Dsg3 was sufficient to fragment sheets equally well in both Ctr and aPKCλ^−/−^ sheets already upon intermediate shear force. Thus, as for control sheets, the increased stability of aPKCλ^−/−^ stratified sheets requires desmosomal adhesion (Fig. 2a; Fig. 2 supplement a,b).

We next asked whether loss of aPKC would alter desmosomal appearance and stained for the desmosomal adhesion receptors Dsg3, Dsg1/2 and the plaque protein desmoplakin. No difference in the punctate distribution representing basal, lateral and apical desmosomes of the lower suprabasal cells, and lateral and basal desmosomes of the most apical suprabasal cells was observed (Fig. 2b). Only in the apical junctional ring of the most apical suprabasal cells Dsg3, Dsg1/2 and desmoplakin staining was strongly reduced upon loss of aPKCλ (Fig. 2b). This apical junctional ring is also co-enriched for AJs (β-catenin, Fig. 2b), and mechanically supports the formation of TJs^23^. Western blot analysis revealed a slight but non-significant reduction in Dsg3 whereas Dsg1 levels were unchanged (Fig. 2 supplement b–d). Together, these data indicate that the increased resilience observed upon loss of aPKCλ did not result from increased expression and/or intercellular recruitment of the desmosomal cadherins.

Desmosomes can take on a hyperadhesive state that enhances epidermal mechanical stability, which is induced by prolonged keratinocyte differentiation^29^. We thus hypothesized that loss of aPKC promotes mechanical resilience through induction of this desmosomal hyperadhesive state. Unlike regular desmosomes, hyperadhesive desmosomes are Ca^2+^-independent and thus remain intact upon Ca^2+^ withdrawal. However, Ca^2+^ chelation was sufficient to induce fragmentation of both aPKC-deficient and control sheets upon mild shear stress, indicating that aPKC does not regulate desmosomal hyperadhesion (Fig. 2c). In agreement, no change in desmoplakin Ser2849 phosphorylation, a marker for hyperadhesion^30^, was observed even upon loss of both aPKCs (Fig. 2d; Fig. 2 supplement c). Changes in desmosomal adhesive strength are also correlated with changes in desmosomal structure i.e. length of individual desmosomes and/or their connection to intermediate filaments^18,31^. Moreover, hyperadhesion is characterized by the formation of an electron-dense intercellular midline due to changes in cadherin packing^32^. Surprisingly, no obvious changes in desmosome ultrastructure, length or number of desmosomes were observed in aPKCλ^−/−^ or aPKC^dKO^ sheets compared to control (Fig. 2e–g). Thus, although our data show that desmosomes are essential for mechanical resilience of stratified epithelial sheets, the enhanced stability observed upon loss of aPKC cannot be explained by the induction of desmosome hyperadhesion nor by increased desmosomal recruitment to sites of cell–cell contact or changes in desmosome ultrastructure.

We then asked whether changes in AJ might explain the increased resilience. Staining for the AJ component β-catenin revealed a strong reduction in intensity and showed a zipper-like instead of linear configuration of AJ in the apical junctional ring upon loss of aPKC. These zippers represent early or immature AJ and require actin reorganization to linearize (Fig. 2b)^22^. However, deleting E-cadherin, the key adhesive AJ protein in these zippers^16^ did not increase fragmentation (Fig. 2h), suggesting that the observed changes in AJs upon loss of aPKC cannot explain the increased mechanical resilience.

### Loss of aPKC regulates keratin filament organization

**Figure 3:**
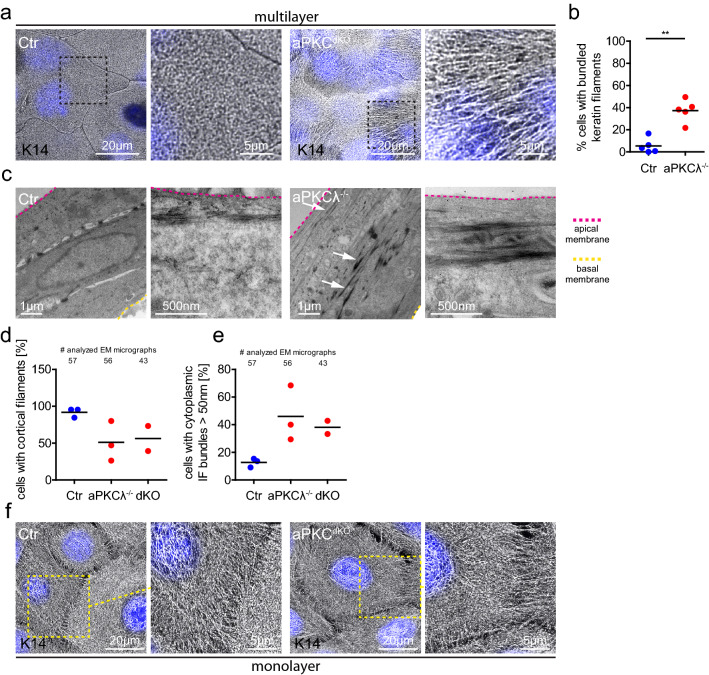
aPKC regulates keratin filament organization. (**a**) Immunofluorescence analysis for keratin14 (K14) in apical cells of stratified keratinocyte cultures (48 h Ca^2+^). Nuclei labeled with DAPI (blue). (**b**) Quantification of cells with bundled keratin fibers. *P* = 0.0079, Mann–Whitney test, Ctr/aPKCλ^−/−^: *n* = 5. Dots represent biological replicates/mice. (**c**) Transmission electron micrographs, cross section of stratified keratinocyte sheets (48 h Ca^2+^) showing strong keratin bundles (white arrows) in suprabasal layers of aPKCλ^−/−^ keratinocytes. Basal lamina is marked by the yellow dashed line. Representative images from n = 3 (Ctr/aPKCλ^−/−^). (**d**) Quantification of apical cells with cortical keratin filaments from EM images as shown in (**c**). (**e**) Quantification of apical cells with thick cytoplasmic keratin filaments from EM images as shown in (**c**). Dots represent biological replicates/mice. (**f**) Immunofluorescence analysis for K14 in keratinocyte monolayers (6 h Ca^2+^). Nuclei labeled with DAPI (blue). Representative example of > 3 biological replicates.

The keratin cytoskeletal network is a further determinant of mechanical stress resistance of epithelial tissues^15^. We thus examined how loss of aPKC affects keratin organization in stratified keratinocytes. Immunofluorescence microscopy revealed that stratified keratinocytes formed a highly organized mesh-like keratin network spanning the entire cell surface (Fig. 3a). Surprisingly, over 40% of stratified aPKCλ^−/−^ or aPKC^dKO^ cells showed long and thick keratin bundles (Fig. 3b) reminiscent of strained keratin filaments^33^, whereas these structures were absent in control cells. In agreement, transmission electron microscopy demonstrated that in > 90% of control keratinocytes cortical keratin filaments run in close proximity to and parallel with the plasma membrane, whereas only about 50% of aPKCλ^−/−^ or aPKC^dKO^ cells showed a membrane-associated keratin network (Fig. 3c,d; Fig. 3 supplement a). Instead, in 40–50% of aPKCλ^−/−^ or aPKC^dKO^ cells keratins were organized in thick (> 50 nm) cytoplasmic bundles compared to only ~ 13% of Ctr cells (Fig. 3c white arrows, e). 3D-TEM tomography confirmed the presence of thick intracellular keratin bundles upon loss of aPKCλ (Supplementary Videos 2, 3). Consistent with the observation that loss of aPKC did not affect basal keratinocyte mechanical resilience (Fig. 1f), the global keratin network organization was not obviously changed upon loss of aPKC (Fig. 3f). Only keratin filaments emanating from the cortex connecting to intercellular junctions appeared elongated (Fig. 3f). Thus, our results show that loss of aPKC promotes bundling and cytoplasmic relocalization of keratins in suprabasal keratinocytes. As keratin organization is an essential determinant of mechanical stress resistance^15^, this increased bundling is likely responsible for increased supracellular mechanical stability.

### Coordinated changes in actomyosin and keratin

**Figure 4:**
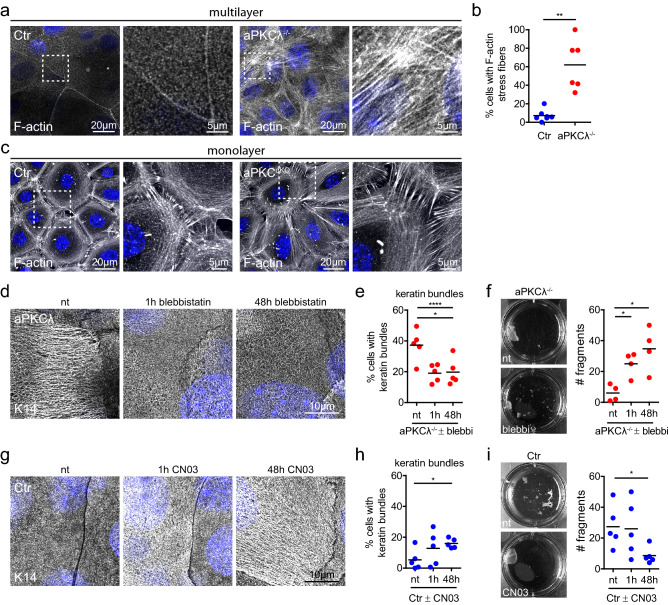
Actomyosin contractility determines keratin bundling and resilience. (**a**) Immunofluorescence analysis for F-actin in stratified keratinocyte cultures (48 h Ca^2+^). Nuclei labeled with DAPI (blue). (**b**) Quantification of cells with F-actin stress fibers. *P* = 0.0012, Mann–Whitney test, Ctr: *n* = 7, aPKCλ^−/−^: *n* = 6. Dots represent biological replicates. (**c**) Immunofluorescence analysis for F-actin in keratinocyte monolayers (6 h Ca^2+^). Nuclei labeled with DAPI (blue). Representative example of > 3 biological replicates. (**d**) Immunofluorescence analysis of keratin14 (K14) in aPKCλ^−/−^ keratinocytes upon inhibition of actomyosin contractility (blebbistatin), treated for either 48 h or 1 h prior to fixation. (**e**) Quantification of cells with bundled keratin filaments as shown in d. *P < 0.05 with 1-way ANOVA, Tukey´s post test for Ctr/aPKCλ^−/−^ each condition *n* = 5. (**f**) dispase assay of stratified aPKCλ^−/−^ sheets (48 h Ca^2+^) and quantification of sheet fragments after sonication upon blebbistatin treatment (5 µM) for indicated timepoints prior to dispase treatment. *P < 0.05 with Mann–Whitney for *n* = 4. (**g**) Immunofluorescence analysis of K14 bundling in Ctr keratinocytes upon Rho activation (CN03), treated for either 48 h or 1 h prior to fixation. (**h**) Quantification of cells with stressed appearance of keratin filaments as shown in (**g**). *P < 0.05 with 1-way ANOVA, Tukey´s post test for Ctr/aPKCλ^−/−^ each condition *n* = 5. (**i**) Dispase assay of stratified Ctr sheets (48 h Ca^2+^) and quantification of sheet fragments after sonication upon Rho activator treatment (CN03) for indicated timepoints prior to dispase treatment. *P < 0.05 with Student’s *t*-test for “nt” vs. “48 h”, *n* = 5. Dots represent biological replicates.

As increased keratin filament bundling occurred without an increase in the number of desmosomes, we asked whether these changes in keratin organization were associated with altered actomyosin organization. aPKC is a known regulator of actomyosin organization and contractility^25^, and cross-talk between actin and keratin cytoskeleton network properties have been reported^19,34–36^. Immunofluorescence analysis of F-actin using phalloidin revealed a highly organized cortically enriched F-actin mesh-like network spanning the entire apical cell surface in suprabasal control keratinocytes (Fig. 4a). Upon loss of aPKCλ or of aPKCλ/ζ this mesh-like network was disturbed and associated with an increase in F-actin stress fibers in around 60% of the suprabasal aPKCλ^−/−^ keratinocytes (Fig. 4a,b).

We next examined whether the increase in suprabasal F-actin stress fibers altered actomyosin contractile behavior of stratified keratinocytes. Dispase treatment to detach the intact keratinocyte sheet resulted in early contraction of the detached sheet, which was completely inhibited by the myosin II inhibitor blebbistatin (Fig. 4 supplement a,b; Supplementary Video 4), indicating that the tissue is under contractile pre-stress that is released upon matrix detachment. Importantly, loss of aPKC increased contractility resulting in earlier detachment from the matrix, which was reversed by blebbistatin (Fig. 4 supplement b,c). We then asked whether changes in contractility were specific for suprabasal keratinocytes. After initiation of intercellular adhesion, F-actin was organized in concentric rings associated with radial fibers connecting to early zipper-like junctions in monolayered keratinocytes as described^22^. In the absence of aPKC, formation of the concentric rings was somewhat impaired with more remaining stress fibers (Fig. 4c). Similar to keratin the radial F-actin connecting to intercellular junctions appeared elongated (Fig. 4c). However, loss of aPKC did not alter monolayer sheet contraction and detachment time (Fig. 4 supplement d). Thus, aPKC controls cellular position dependent F-actin reorganization and contractile pre-stress state of suprabasal cells in stratified epidermal sheets.

### Actomyosin network controls keratin organization and mechanical resilience

We next hypothesized that keratins reorganize into bundles and increase mechanical resistance to protect tissue integrity in response to enhanced suprabasal actomyosin contractility induced by loss of aPKC. To directly test this hypothesis, we lowered contractility in aPKCλ^−/−^ stratified keratinocytes using low doses of blebbistatin (5 µM) (Fig. 4 supplement e). Reducing contractility was sufficient to significantly lower the number of cells with bundled keratin filaments in aPKCλ^−/−^ sheets, even upon short-term inhibition (Fig. 4d,e). Thus, the increased contractility induced by loss of aPKC is responsible for the increase in keratin bundling. Importantly, low doses of blebbistatin also reduced the resilience of stratified keratinocyte sheets, thus directly linking the changes in cytoskeletal organization and activity with mechanical stability (Fig. 4f).

To test whether induction of actomyosin contractility is sufficient to induce keratin bundling and increase resilience in control stratified sheets, we next increased contractility using the Rho-activator CN03. Both short-term CN03 treatment of differentiated sheets (1 h, 47–48 h Ca^2+^), and continuous treatment throughout the differentiation process (0–48 h Ca^2+^) strongly increased the percentage of stratified keratinocytes with keratin bundles (Fig. 4g,h; Fig. 4 supplement f), and promoted mechanical stability (Fig. 4i). Importantly, most cells with stressed keratin bundles also had F-actin stress fibers (~ 89%) (Suppl. Fig. 4g,h), indicating a cell autonomous response of keratins to the inability to reorganize actomyosin upon stratification. Thus, the contractile state of the suprabasal actomyosin cytoskeleton determines the structure and mechanical properties of the keratin network.

Taken together, these data show that aPKC controls the reorganization of the actomyosin network to reduce suprabasal contractility in stratified keratinocytes. Moreover, a failure in actomyosin reorganization is sensed by the keratin filament system resulting in adaption of its organization that likely mediates the increase in mechanical resilience to maintain tissue integrity in the face of prestress.

### aPKC regulates cytoskeletal reorganization in vivo

**Figure 5:**
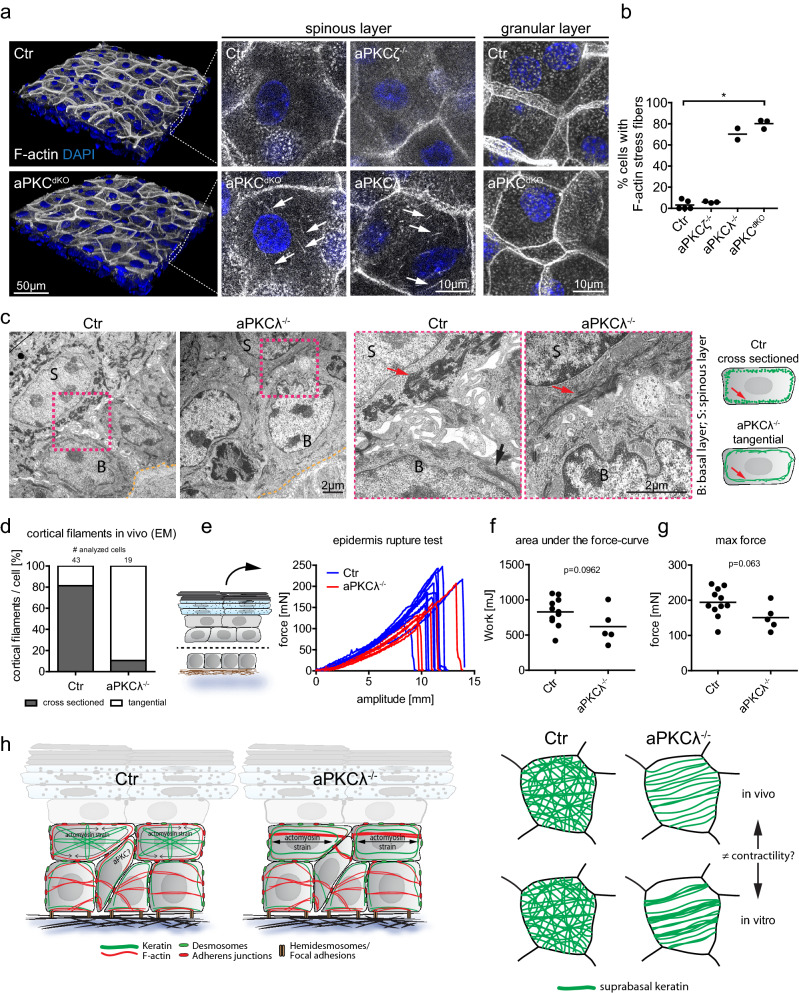
aPKC regulates cytoskeletal reorganization and tissue resilience in vivo. (**a**) Newborn mouse epidermal whole-mount immunofluorescence analysis for F-actin revealing stress fibers (white arrows) in the first suprabasal layers upon epidermal loss of aPKCλ (aPKCλ^−/−^) or both aPKCλ and ζ (aPKC^dKO^) but not in Ctr or aPKCζ epidermal knockout (aPKCζ^−/−^) mice. (**b**) Quantification of cells with F-actin stress fibers in suprabasal (spinous) layers. Each dot represents one mouse. *P* = 0.0286 with Kruskal–Wallis, Dunn’s post hoc test for Ctr: *n* = 5, aPKCζ: *n* = 3, aPKCλ: *n* = 2, aPKC^dKO^: *n* = 3. (**c**) Transmission electron micrographs, cross section of newborn mouse epidermis showing an altered orientation of keratin bundles (red arrows) in suprabasal (spinous) layers of epidermal aPKCλ^−/−^ mice. Basal lamina is marked by the yellow dashed line. Representative images from n = 4 (Ctr) and n = 3 (aPKCλ) mice. (**d**) Quantification of spinous cells with regular (cross sectioned) and irregular (tangentially cut or absent) keratin bundles. Cumulative values from n = 4 (Ctr) and n = 3 (aPKCλ) mice. (**e**) Measured force over time while stretching isolated suprabasal epidermis. (**f**) Quantification of the area under the curve from e. With Student´s *t*-test for n = 11 (Ctr) and n = 5 (aPKCλ^epi−/−^). (**g**) Quantification of the maximum force that was reached during stretching, prior to sheet rupture. With Student’s *t*-test for n = 11 (Ctr) and n = 5 (aPKCλ^epi−/−^). Dots represent biological replicates. (**h**) Model of aPKC dependent suprabasal intermediate filament organization.

We then asked whether aPKC also controls keratin and actin cytoskeletal (re-)organization in suprabasal cells in vivo. 3D epidermal whole mount imaging of newborn mouse epidermis showed that cells of the first suprabasal spinous layer showed aberrant cytoplasmic F-actin fibers upon loss of aPKCλ or both isoforms (Fig. 5a,b), even if cortical F-actin networks still form, thus indicating increased suprabasal contractile prestress also in vivo. Thus, aPKC also controls the in vivo reorganization of actin filaments when cells move suprabasally into the spinous layer.

Ultrastructural analysis revealed that when sectioned in the plane of the nucleus basal control cells in vivo contain tangentially sectioned keratin bundles (Fig. 5c, black arrow) whereas the above suprabasal spinous layer showed the presence of cross-sectioned submembrane keratin bundles (~ 80% of cells) (Fig. 5c, red arrow). Thus, in vivo basal versus suprabasal cells have a different cellular organization of the K5/K14 versus K1/K10 network, respectively. Previously we had shown that epidermal loss of aPKCλ does not obviously affect the basal to suprabasal shift in keratin subtypes^37^. However, upon loss of aPKCλ about 90% of spinous cells showed tangentially sectioned keratin bundles, suggesting that aPKC is required to organize K1/K10 networks in suprabasal keratinocytes in vivo (Fig. 5c,d,h; Fig. 5 supplement a). Surprisingly, aPKC-deficient keratinocytes did not show increased keratin bundling (Fig. 5c). Thus, in vivo as in vitro changes in suprabasal keratin network organization are associated with the presence of suprabasal actin stress fibers and increased contractile stress.

We then asked whether these changes in keratin organization induced by loss of aPKC regulates suprabasal epidermal resilience. To this end, we isolated and separated the skin between the basal and first suprabasal layer (Fig. 5e) and used the micro-tissue stretcher to subject the suprabasal epidermis (all suprabasal layers) to pulling forces until rupture. In contrast to in vitro, there was no statistically significant difference between aPKCλ^−/−^ and Ctr epidermis (Fig. 5e–g), in the resistance forces even if there was a tendency for a small reduction upon loss of aPKC (1.33 fold reduced, area under the curve) (Fig. 5e–g). Thus, despite indications of increased contractility the mechanical resilience of suprabasal layers is largely preserved upon loss of aPKC associated with changes in suprabasal keratin organization.

Together, these results indicate that aPKC in vitro and in vivo coordinates the reorganization and contractile state of the actomyosin network and that keratins can sense changes in suprabasal prestress and adapt their network organization to preserve mechanical stability of the epidermis (Fig. 5h).

## Discussion

The extraordinary resilience of the skin epidermis to mechanical stress has been mostly attributed to the desmosome-keratin intermediate filament (IF) system^2,8,38,39^. How resilience is regulated in the face of dynamic cellular rearrangements is not well understood. Here we show that aPKC reorganizes suprabasal actomyosin stress fibers into a membrane-associated F-actin network during stratification. In the absence of aPKC, suprabasal keratinocytes fail to reorganize actin, resulting in increased suprabasal contractile stress. In response to this aberrant contractility, suprabasal keratins reorganize their network associated with strengthened cellular mechanical resilience that through desmosomes is communicated supracellularly, thus maintaining tissue integrity. Our data thus suggest an important role for keratins in sensing of and mechanical adaption to actomyosin prestress.

Previous studies have shown an intimate connection between epithelial actin and keratin networks. For example in in vitro reconstituted networks of F-actin and keratin, F-actin serves as a steric hindrance to prevent keratin network collapse^34^. In simple epithelia F-actin also regulates retrograde transport of keratin subunits to promote their integration into the keratin network^35^, whereas keratins can reinforce F-actin stress fibers in these cells through interaction with the Rho-GEF Solo^40^. In keratinocytes keratin filaments closely associate with actin stress fibers before and during initial formation of intercellular junctions^41^. Thus, keratin and actin crosstalk regulates their respective network organization. Our data now provide evidence that the inability to lower contractility through reorganization of F-actin in suprabasal differentiated cells triggers a mechano-adaptive response of the keratin network.

How keratins change network structure upon inappropriate suprabasal F-actin organization and tension is currently not clear. Interestingly, F-actin and keratin filaments do not substantially align (Fig. 4 supplement g), suggesting that F-actin organization is not simply used as a blueprint for keratin organization. A more likely explanation is that actomyosin and keratin networks form a viscoelastic composite to balance pre-stress and mechanoresistance of the epidermal sheet. This hypothesis is supported by studies showing that the viscoelastic properties of these composite filament networks is in part determined by the ratio of F-actin and keratin within these networks^42^. The inability to reorganize F-actin in combination with differentiation-dependent changes in the densities of the networks may thus affect the F-actin/keratin ratio, thereby changing the mechanical properties of the cell sheet. One remaining caveat of our study is that we cannot directly show that keratins are causal for the increase in mechanical resilience as currently no drugs or other manipulations exist to directly interfere or strengthen keratin network organization. Regardless of the precise mechanism, lowering the increased contractility in the absence of aPKC normalizes both keratin network organization and mechanical resilience whereas increasing contractility in Ctr is sufficient to change keratin network reorganization and promote sheet resilience, similar to what we observe upon loss of aPKC. Together, these data demonstrate that the contractile state of the stratified keratinocyte sheet is an important determinant for the keratin adaptive response.

How suprabasal actomyosin prestress is communicated on the molecular level to induce keratin bundling and mechanical strengthening remains a key open question. One possibility is that stress alters the keratin phosphorylation state, which regulates keratin bundling^43^. Alternatively, keratin elongation and cycling could be altered through for example a change in keratin transdimer disulfide bond formation^44^. A third possibility is altered communication through molecular players like the plakin protein plectin that link F-actin and keratin networks^45^. Interestingly, loss of plectin induced increased contractility in epithelial cells^45–47^ similar to loss of aPKC, but resulted in decreased and not increased sheet resilience. Thus, plectin might reorganize keratin to counteract the increased suprabasal contractility induced by loss of aPKC.

Loss of aPKC enhanced resilience in vitro whereas in vivo resilience was not significantly altered, suggesting that in both cases suprabasal cells counteract contractile stress but to a different extent to preserve mechanical tissue integrity. The reason for this phenotypic difference is not fully clear. Increased keratin bundling was only observed in vitro and not in vivo, which might explain the enhanced in vitro response. Whereas keratinocytes switch from a K5/K14 to a K1/K10 network when moving suprabasally, cultured stratifying mouse keratinocytes fail to properly induce K1/K10. The quality of the keratin network response to the actomyosin state may thus depend on their isotype. Even if the in vivo and in vitro mechanical environments are substantially different*,* our data indicate that tissue-scale mechanics are balanced through aPKCλ. Importantly, proper balancing suprabasal contractile activity is likely physiological relevant as too much mechanical resilience may impair the ability of the epidermis to efficiently renew and repair.

Our data identify aPKC as an important regulator of actomyosin network dynamics and reorganization during the formation of multilayered epithelial sheets in vitro^22,23^ and in the transition from basal to spinous layer in vivo. As aPKC regulates AJ states, the local activation level of aPKC may coordinate cortical and junctional plasticity that is essential to enable cellular rearrangements in the formation and turnover of stratified epithelia. Consistently, loss of aPKCλ affects skin wound healing^48^ whereas overexpression of aPKCλ induces cell extrusion from mammary epithelium^26^, both processes that depend on cellular rearrangements. Together, these data thus suggest that aPKC is a rheostat for actomyosin and junctional mechanics that enable stratified epithelial tissue formation and regeneration.

In conclusion, our data uncovered an important role for aPKC in coordinating cytoskeletal network transitions and identify a novel keratin-dependent adaptive mechanism how tissues maintain integrity in the face of increased mechanical stress.

## Materials and methods

### Mice

aPKCζ total body knock out mice and epidermal aPKCλ deletion were described before^37,49^. aPKCλ epidermal specific knock out mice were generated by crossing aPKCλ-floxed mice with Keratin-14 promoter driven Cre expressing mice^50^. Epidermal specific deletion of both aPKC isoforms were achieved by crossing aPKCζ total body knock out mice with epidermis specific aPKCλ knock out mice.

### Isolation and culture of primary keratinocytes

Primary keratinocytes were isolated and cultured as described before^23^: newborn mice were decapitated and incubated in 50% betaisodona/PBS for 30 min at 4 °C, 1 min PBS, 1 min 70% EtOH, 1 min PBS and 1 min antibiotic/antimycotic solution. Tail and legs were removed and complete skin incubated in 2 ml dispase (5 mg ml^−1^ in culture medium) solution. After incubation over night at 4 °C, skin was transferred onto 500 µl FAD medium on a 6 cm dish and epidermis was separated from the dermis as a sheet. Epidermis was transferred dermal side down onto 500 µl of TrypLE (Thermo Fisher Scientific) and incubated for 20 min at RT. Keratinocytes were washed out of the epidermal sheet using 3 ml of 10% FCS/PBS. After centrifugation keratinocytes were resuspended in FAD medium and seeded onto collagen type-1 (0.04 mg ml^−1^) (Biochrom, L7213) coated cell culture plates. Primary murine keratinocytes were kept at 32 °C and 5% CO_2_.

Primary keratinocytes were cultured in DMEM/HAM’s F12 (FAD) medium with low Ca^2+^ (50 μM) (Biochrom) supplemented with 10% FCS (chelated), penicillin (100 U ml^−1^), streptomycin (100 μg ml^−1^, Biochrom A2212), adenine (1.8 × 10^−4^ M, SIGMA A3159), l-glutamine (2 mM, Biochrom K0282), hydrocortisone (0.5 μg ml^−1^, Sigma H4001), EGF (10 ng ml^−1^, Sigma E9644), cholera enterotoxin (0.1 nM, Sigma C-8052), insulin (5 μg ml^−1^, Sigma I1882), and ascorbic acid (0.05 mg ml^−1^, Sigma A4034).

### Transfection

Overexpression: Keratinocytes were transfected at 100% confluency with Viromer®Red. 1.5 µg DNA were diluted in 100 µl Buffer E, added to 1.25 µl Viromer®RED and incubated for 15 min at RT^23^. 33 µl transfection mix were used per well with 0.5 ml FAD medium (24 well plate). Knockdown: Keratinocytes were transfected at 100% confluency with Viromer®BLUE (lipocalyx; Halle Germany). Transfection mix was prepared according the manufacturers protocol. 100 µl transfection mix was used per well with 1 ml FAD medium (12 well plate, 5 nM siRNA f.c.). siRNA (siPOOLs) against the 3′ UTR of mouse Dsg3 and against the ORF of mouse Iqgap1 were obtained from (siPOOLs from siTOOLs, Planegg, Germany).

### Immunofluorescence of keratinocytes in vitro

Immunofluorescence stainings of keratinocytes were performed as described before^51^: cells were seeded on collagen coated glass cover slips in a 24 well plate and switched to high Ca^2+^ medium as indicated in the results section.

Cells were fixed using 4% PFA for 10 min at RT, washed three times for 5 min using PBS, permeabilized using 0.5% TritonX100/PBS and blocked using 5% NGS/1% BSA/PBS for 1 h at room temperature. Primary antibodies were diluted as indicated in the antibody section in Background Reducing Antibody Diluent Solution (ADS) (DAKO). Cover slips were placed growth surface down onto a 50 µl drop of staining solution on parafilm in a humidified chamber and incubated overnight at 4 °C. Cover slips were washed again with PBS three times for 10 min. Secondary antibodies and DAPI (40,6-diamidin-2-phenylindol, Sigma) were diluted 1:500 in ADS and cover slips were incubated for 1 h at RT. Secondary antibodies were washed off via three wash steps using PBS for 10 min. Cover slips were mounted using Mowiol (Calbiochem).

### Antibodies and inhibitors

Mouse monoclonal against **Desmoglein1/2** (IF 1:200, Progen #61002); mouse monoclonal against **Desmoglein3** (IF 1:2000, WB 1:1000, MBL #D218-3); mouse monoclonal against **Desmoplakin1/2** (IF 1:200, Progen #61003); rabbit against the c-terminus of human **Desmoplakin1/2** (NW6) (WB: 1:1000, Angst et al., 1990, kind gift from Kathleen J. Green), rabbit against phospho-S2849 of **Desmoplakin** (WB: 1:4000, Bouameur et al., 2013, kind gift from Kathleen J. Green), mouse monoclonal antibody against the cytoplasmic domain of **E-cadherin** (IF 1:200, BD Transduction Laboratories #610182, clone number 36); mouse monoclonal against **GAPDH** (WB 1:10000, Ambion #AM4300); rabbit polyclonal against **Keratin14** (IF 1:2000, Covance #PRB 155P); rabbit monoclonal against **β-catenin** (IF 1:1000, Abcam #ab32572). **Phalloidin** was used to stain F-actin (IF 1:500, Sigma #P1951, TRITC conjugated). Secondary antibodies were species-specific antibodies conjugated with either AlexaFluor 488, 594 or 647, used at a dilution of 1:500 for immunofluorescence (Molecular Probes, Life Technologies), or with horseradish peroxidase antibodies used at 1:5000 for immunoblotting (Bio-Rad Laboratories).

Inhibitors used in this study: Blebbistatin myosin inhibitor, 5 μM (Sigma #B0560); Rho activator, 5 µg/ml (Cytoskeleton #CN03).

### Preparation of epidermal whole mounts

Epidermal whole mounts were prepared as has been described previously^23^.

### Sheet integrity assay

1.5 million Ctr or knockout keratinocytes were seeded on a 6 well (or 0.5 million/12 well). One day after seeding, medium was switched to high Ca^2+^ medium for 48 h. To detach sheets from the plate, sheets were incubated with dispase containing high Ca^2+^ medium (5 mg ml^−1^ dispase) for about 30 min until sheets detached. Dispase medium was removed and sheet washed 2× with PBS supplemented with 1 mM Ca^2+^. **Mild shear stress**: sheets were subjected to mild stress using rotation (150 rpm) on an orbital shaker in 2 ml PBS/Ca^2+^ for 15 min. If no fragmentation was observed, **intermediate shear stress** was applied: sheets were transferred to a 15 ml falcon containing 5 ml PBS/Ca^2+^ and rotated end over end for 5 min. If no fragmentation was observed, **high shear stress** was applied: sheets were subjected to sonication by inserting a sonication probe in the tube used for rotation before. Sheets were sonicated 1–3 times for 1 s/10% amplitude (Branson ltd. Digital Sonifier 250CE). For EGTA treatment, sheets were washed 2× with PBS/3 mM EGTA and subjected to mild stress using rotation (150 rpm) on an orbital shaker in 2 ml PBS/3 mM EGTA for 1 h. Sheet fragments were imaged with a BIO RAD Gel Doc system and counted with Fiji^52^ using color thresholding to detect fragments and the analyze particle tool for counting.

### Tissue stretching

Stratified keratinocyte sheets from 6 well plates were prepared as described for the sheet integrity assay. Keratinocyte sheets were then treated as described before^28^: sheets were transferred to a custom-made tissue stretcher, by placing them on a C-shaped PVDF-membrane frame, which served as carrier and distance piece. Together they were mounted in the holding frame. The lateral ligament of the PVDF frame allowed accurate length maintenance of the specimen. After mounting, the frame was cut and the sample placed in the PBS-filled chamber. The starting sample length was set to 10 mm using a single-lens reflex camera (EOS600D, Canon, Japan) equipped with a macro objective (Macro lens EF-S 60 mm, Canon, Japan). Sheet were strained with 20 mm min^−1^ until rupture. The resistive force was recorded throughout the experiment with a sampling frequency of 10 Hz. For details of the stretcher device see also^28^.

### Sheet detachment assay

50,000 cell were seeded/96 well and switched to high Ca^2+^ after 24 h. After 48 h of differentiation in high Ca^2+^ medium, dispase was added (f.c. 1.25 mg ml^−1^ dispase) and detachment of sheets was imaged every 30 s using an Evos system at 32 °C and 5% CO_2_. For inhibition of actin polymerization or actomyosin contractility, 0.1 µM f.c. LatrunculinB or 100 µM f.c. blebbistatin were added 30 min prior to detachment, respectively.

### Microscopy

Confocal images were obtained with a Leica TCS SP8, equipped with gateable hybrid detectors (HyDs). Objectives used with this microscope: PlanApo 63x, 1.4 NA CS2. Images to be used for deconvolution were obtained at optimal resolution according to Nyquist. Epifluorescence images were obtained with a Leica DMI6000. Objectives used with this microscope: PlanApo 63x, 1.4 NA. Sheet detachment assays were imaged using an Evos FL Auto2 (Life Technologies) equipped with a High-sensitivity 1.3 MP CMOS monochrome camera.

### Transmission electron microscopy

Mouse backskin was spread on whatman paper and immersion fixed in 2% formaldehyde, 2% glutaraldehyde in 0,1 M sodium cacodylate buffer (Applichem) for 48 h at 4 °C.

For epon embedding, fixed tissue was washed with 0,1 M sodium cacodylate buffer, incubated with 2%OsO_4_ (Science Services) in 0,1 M cacodylate buffer for 2 h at 4 °C, and washed three times with 0,1 M cacodylate buffer. Subsequently, tissue was dehydrated at 4 °C using ascending ethanol series for 15 min 50%, overnight 70%, 15 min 90%, 3 × 15 min 100%, 15 min 50% ethanol/propylene oxide and 2 × 15 min 100% propylene oxide. Tissue was infiltrated for 2 h with 50% epon in propylene oxide (Sigma Aldrich), 2 h 75% epon in propylene oxide, overnight 100% epon and finally 2 h with fresh epon at RT. Tissue was transferred into embedding moulds and cured for 72 h at 60 °C. Ultrathin sections of 70 nm were cut using an ultramicrotome (Leica Microsystems, UC6) and a diamond knife (Diatome, Biel, Switzerland) and stained with 1.5% uranyl acetate for 15 min at 37 °C and lead citrate solution for 4 min. Images were acquired using a JEM-2100 Plus Transmission Electron Microscope (JEOL) operating at 80 kV equipped with a OneView 4 K camera (Gatan).

### Transmission electron tomography

Ultrathin sections of 200 nm were cut using an ultramicrotome (Leica, UC7) and incubated with 10 nm protein A gold (CMC, Utrecht) diluted 1:20 in ddH20. Sections were stained with Reynolds lead citrate solution for 3 min. Tilt series were acquired from − 65° to 65° with 1° increment on a JEM-2100 Plus Transmission Electron Microscope (JEOL) operating at 200 kV equipped with a OneView 4 K 32 bit (Gatan) using SerialEM^53^. Reconstruction was done using Imod^54^.

### Image analysis

#### Quantification of desmosomes from EM images

Desmosome number and length were manually quantified from low and high magnification images of transmission electron micrographs using the Fiji line tool.

#### Quantification of filaments from IF images

Cells with F-actin stress fibers or keratin bundles were clearly distinguishable from cells with regular cortices and were counted manually from confocal stacks of phalloidin and keratin14 labeled epidermal whole mounts or in vitro stratified sheets.

#### Quantification of cortical filaments from EM images in vitro

Using high magnification images of transmission electron micrographs the two uppermost (most differentiated) cells in each image of stratified keratinocytes (48 h Ca^2+^) were manually categorized for the presence of cortical intermediate filaments and occurrence of cytoplasmic intermediate filament bundles. Examples of quantified categories are given in Fig. 3 supplement a. The number of analyzed EM images is displayed above the respective graphs.

#### Quantification of cortical filaments from EM images in vivo

Using high magnification images of transmission electron micrographs, spinous layer cells that were cut through the nucleus (as a reference for sectional plane position) were manually categorized for the presence of regular cortical cross sectioned intermediate filaments bundles as shown in Fig. 5c. Spinous cells of aPKCλ deficient epidermis at this sectional plane, show tangentially cut filament bundles as shown in Fig. 5c. The number of analyzed cells is displayed above the respective graphs.

### Statistics and repeatability of experiments

The numbers of independent experiments and biological replicates performed for all experiments, *p* values and the statistical tests that were used are indicated in the figure legends.

### Ethics declaration

All animal protocols in this study have been approved by the animal experiment committee of LANUV, North Rhine-Westphalia, Germany. All methods were carried out in accordance with relevant guidelines and regulations. The animal protocols and the reporting in this manuscript follow the recommendations in the ARRIVE guidelines.

## Supplementary Information

Supplementary Information.

Supplementary Video 1.

Supplementary Video 2.

Supplementary Video 3.

Supplementary Video 4.

## Data Availability

The authors declare that the data supporting the findings of this study are available within the paper and its Supplementary Information files. Additional data are available from the corresponding author upon reasonable request.
