# Supplementary material for: Polarity signaling balances epithelial contractility and mechanical resistance
Source: Sci Rep. 2023 May 12;13:7743. doi: 10.1038/s41598-023-33485-5 (PMC10182030; doi:10.1038/s41598-023-33485-5)
Supplement: Supplementary file 1 — Supplementary Information. [file 41598_2023_33485_MOESM1_ESM.docx]

**Polarity signaling balances epithelial contractility and mechanical resistance**

Matthias Rübsam, Robin Püllen, Frederik Tellkamp, Alessandra Bianco, Marc Pescoller, Wilhelm Bloch, Kathleen J. Green, Rudolf Merkel, Bernd Hoffmann, Sara A. Wickström and Carien M. Niessen

Supplementary information

**
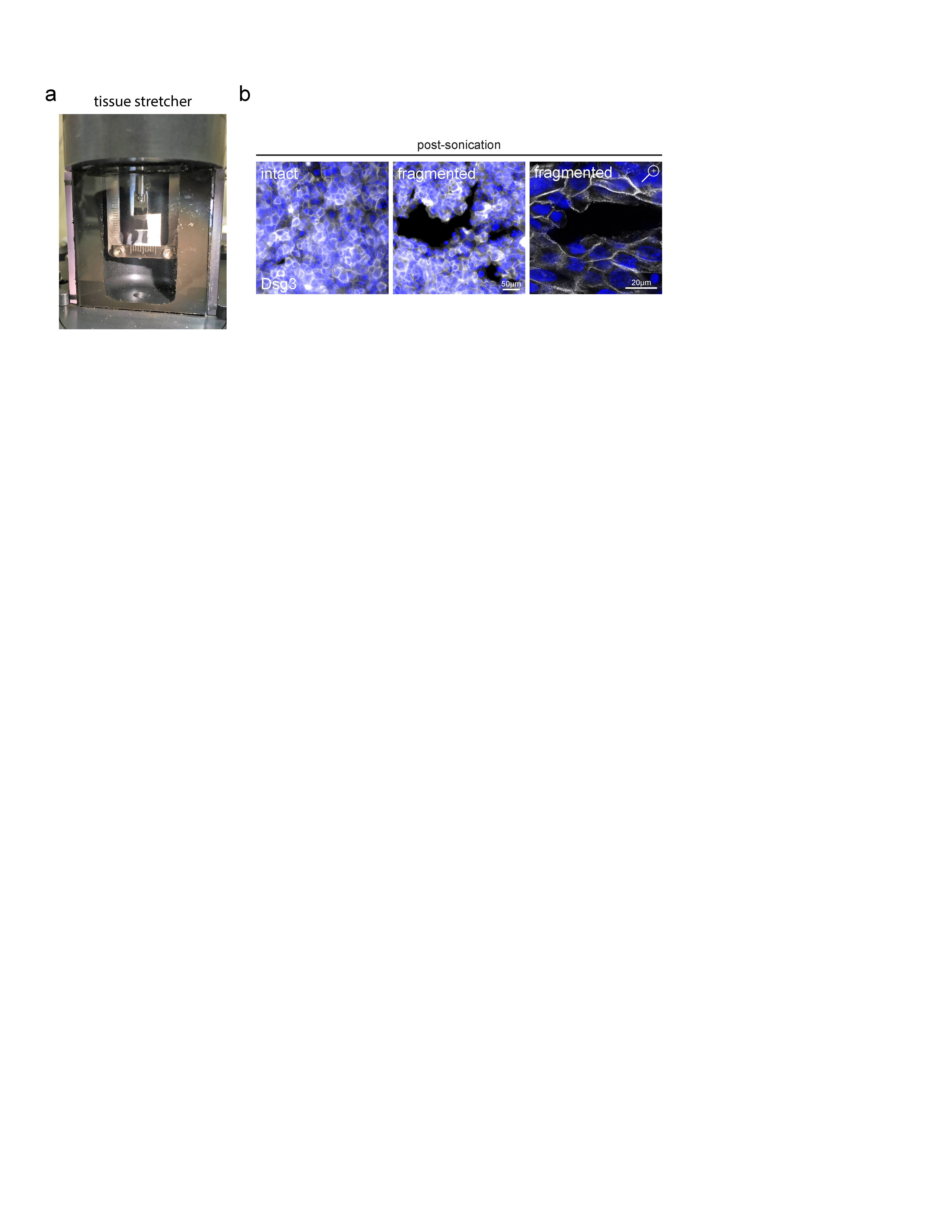
**

**Fig. 1 supplement. a** Measuring chamber of the tissue stretcher. **b** Staining for intercellular contacts by desmoglein3 (Dsg3) in fragmented keratinocyte sheets post sonication showing intact cellular structure with inter- or intracellular rupture.

**
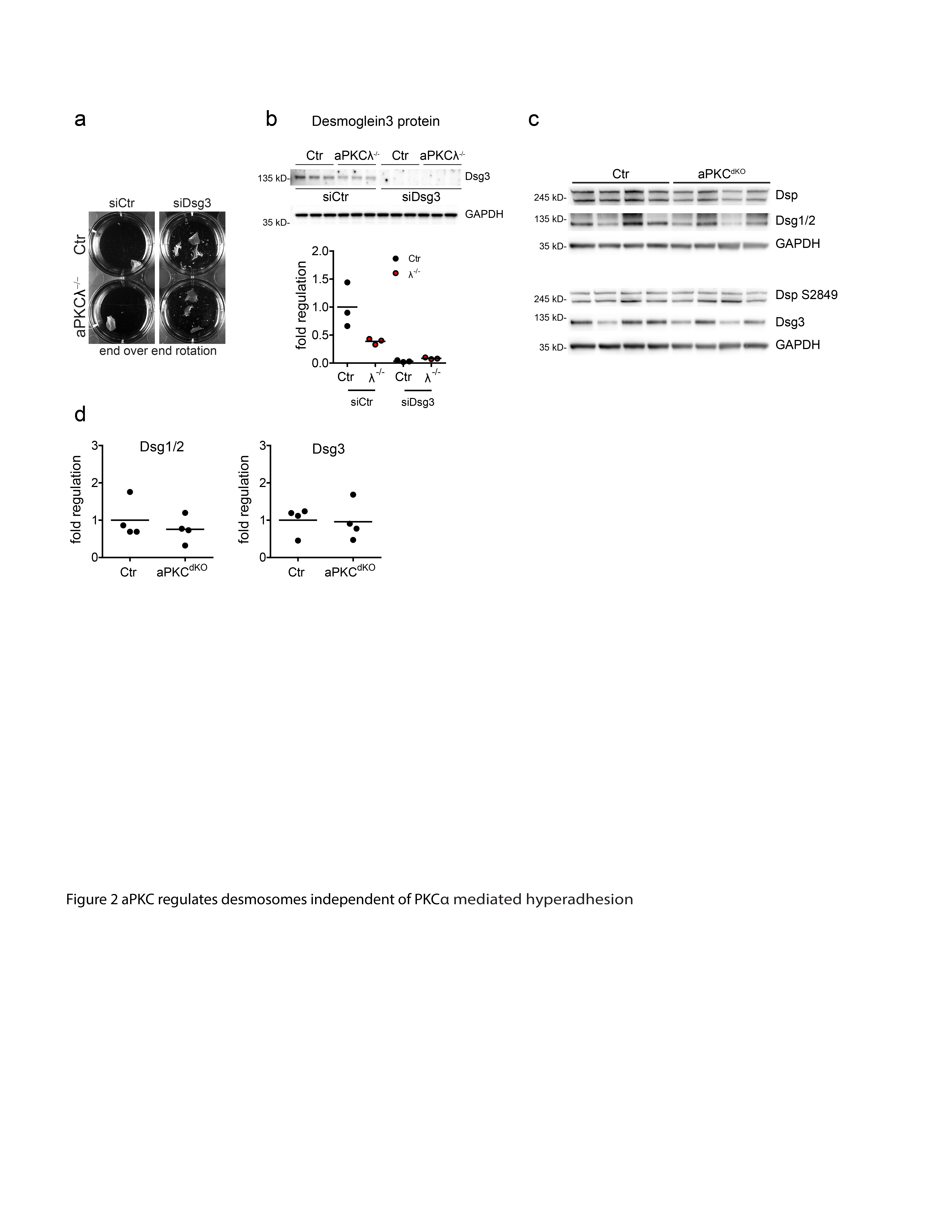
**

**Fig. 2 supplement. a** Representative dispase assay upon knockdown of desmoglein3 (siDsg3) in Ctr or aPKCλ^-/-^ stratified keratinocyte sheets (48 h Ca^2+^). Examples of sheets after end over end rotation. **b** Western blot (cropped and straightened) and quantification of desmsoglein3 protein levels in Ctr and aPKCλ^-/-^ keratinocytes and upon transfection with Dsg3 specific siRNA pools. Lysates from stratified sheets (48 h Ca^2+^) post dispase assay. Original blots are presented in Fig. 2 supplement 2 a. **c** Representative western blot (cropped and straightened) for total protein levels of Dsg1/2, Dsg3 and total and phospho-desmoplakin (Ser2849) in lysates of stratified keratinocyte sheets (48 h Ca^2+^). Original blots are presented in Fig. 2 supplement 2 b. **d** Western Blot quantification of total Dsg1/2 and Dsg3 protein levels in lysates of stratified keratinocyte sheets (48 h Ca^2+^).

**
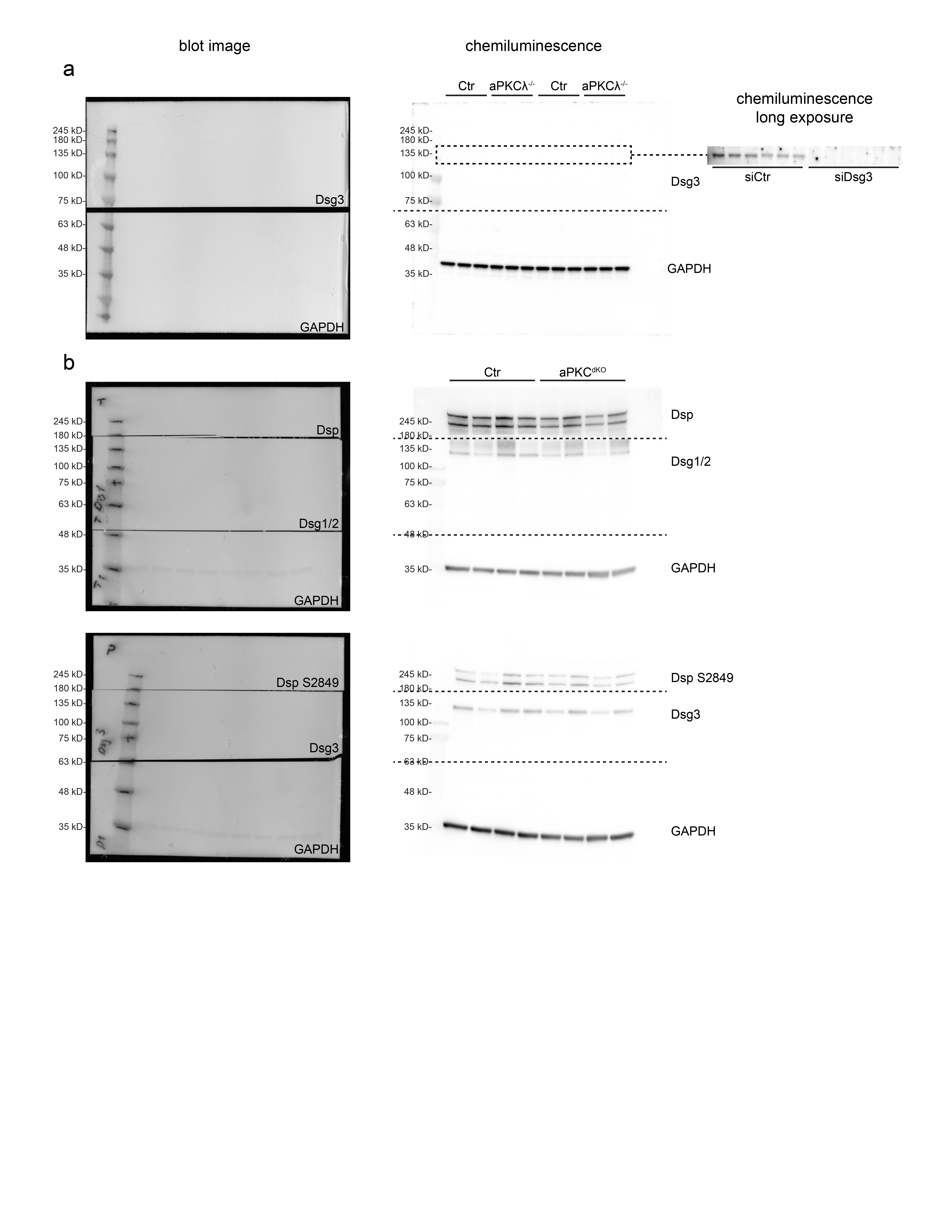
**

**Fig. 2 supplement 2**. **a** Original blots corresponding to Fig. 2 supplement b. **b** Original blots corresponding to Fig. 2 supplement c.

**
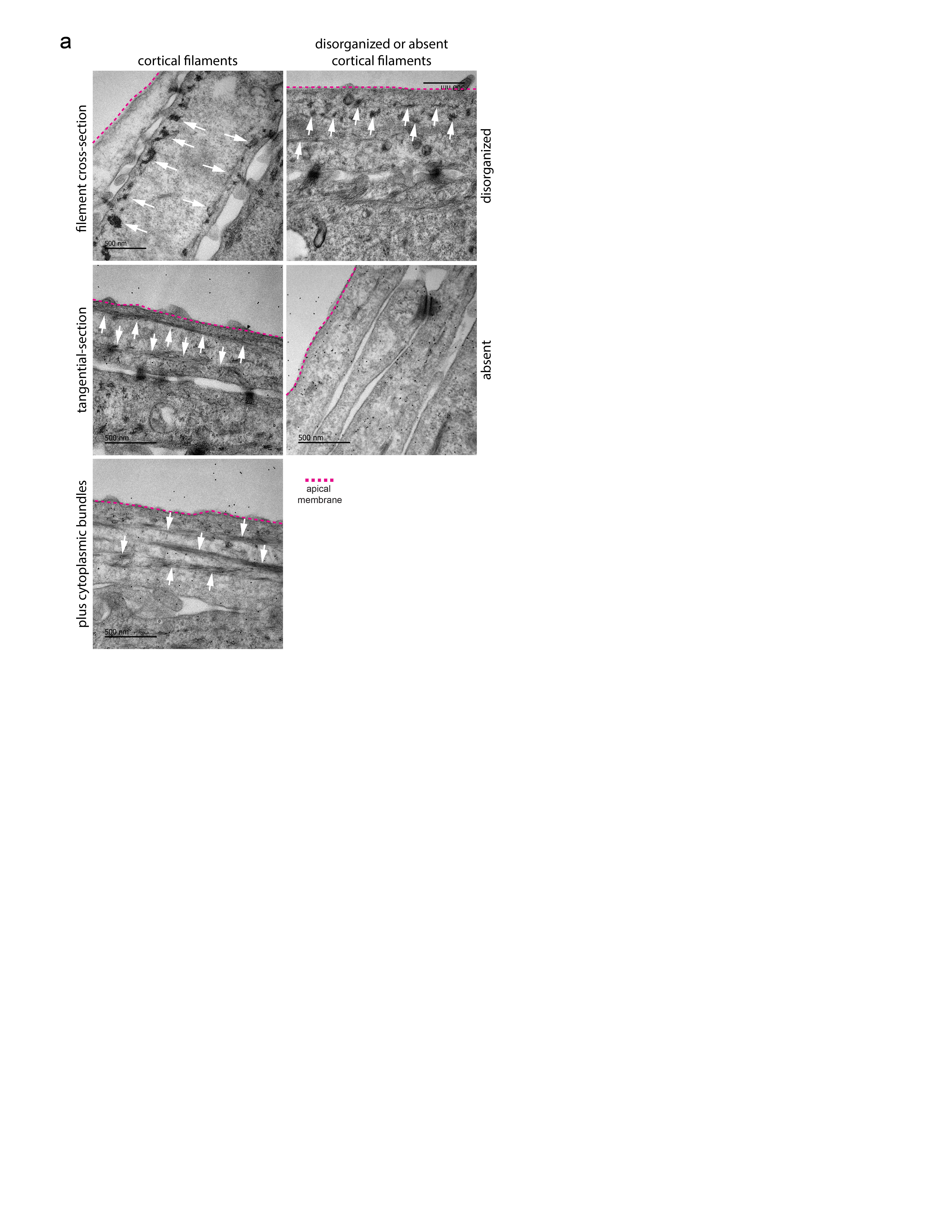
**

**Fig. 3 supplement a** Transmission electron micrographs, cross section of stratified keratinocyte sheets (48 h Ca^2+^). Examples of quantified categories for the presence or absence of cortical keratins and cytoplasmic keratin bundles.


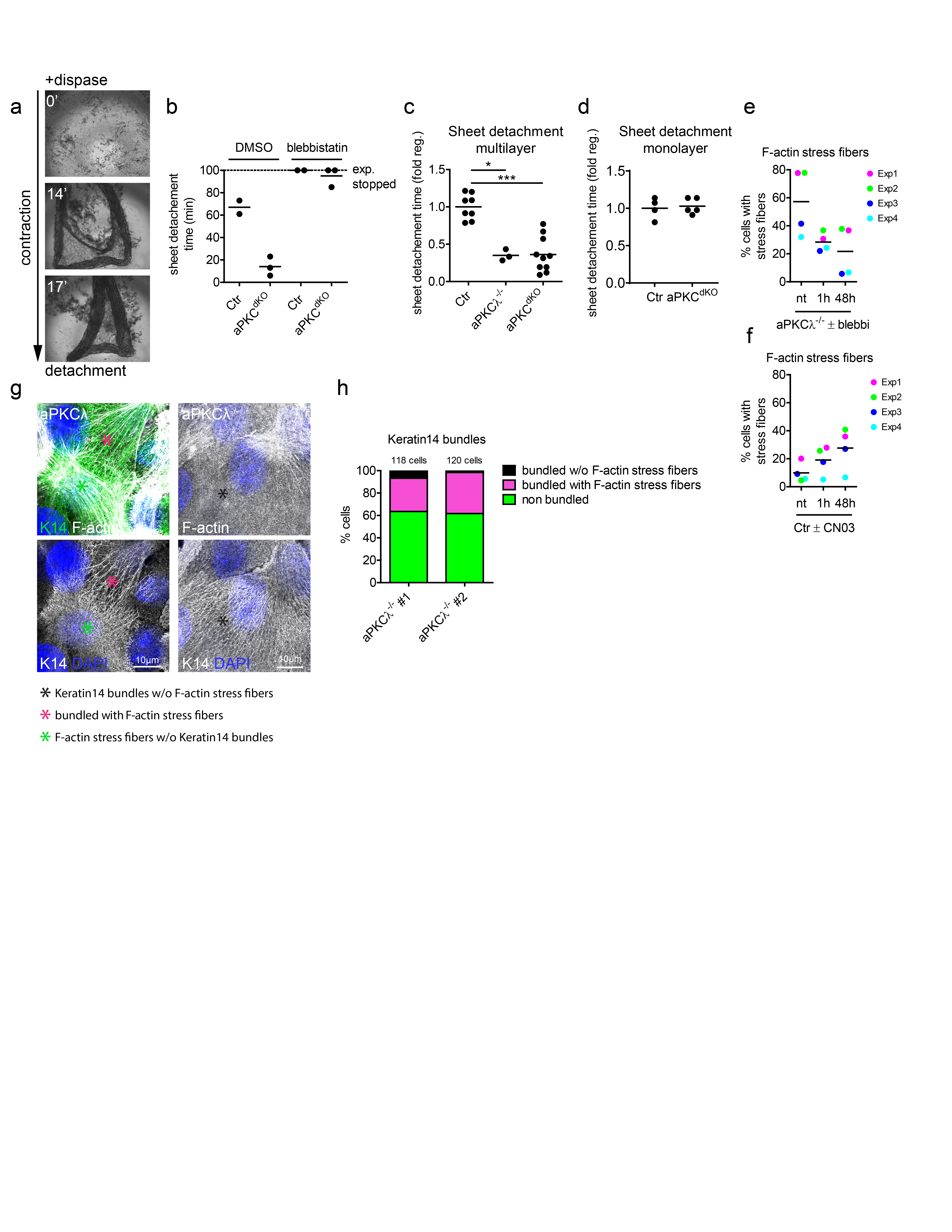


**Fig. 4 supplement. a** Stills (0, 14, 17 minutes) from live imaging of multilayered keratinocyte sheets upon dispase treatment showing sheet contraction. **b, c** Quantification of multilayer sheet detachment time upon dispase treatment in the presence or absence of 20 µM blebbistatin (**b**) and of Ctr, aPKCλ^-/-^ and aPKC^dKO^ sheets (**c**). **P* < 0.05 with Kruskal–Wallis, Dunn’s post test for Ctr: *n*=8, aPKCλ^-/-^: *n*=3, aPKC^dKO^: *n*=10 (**c**). **d** Quantification of monolayer sheet detachment time upon dispase treatment of Ctr and aPKC^dKO^ sheets (Ctr: *n*=4, aPKC^dKO^: *n*=5. **e, f** Quantification of cells with F-actin stress fibers in Ctr keratinocytes upon Rho activation (CN03) and aPKCλ^-/-^ keratinocytes upon blebbistatin treatment (5 µM) for indicated timepoints prior to fixation. Samples (dots) of one experiment are labeled in one color to demonstrate consistent up- or downregulation of F-actin stress fibers upon treatment despite varying base levels. **g** Immunofluorescence analysis for colocalization of F-actin stress fibers and bundled keratin14 (K14) in apical cells of stratified aPKCλ^-/-^ keratinocytes (48 h Ca^2+^). **h** Quantification of cells with keratin bundles with respect to F-actin stress fibers in the same cell. Two biological replicates of stratified aPKCλ^-/-^ keratinocytes are shown. Numbers of total quantified cells are indicated above bars.


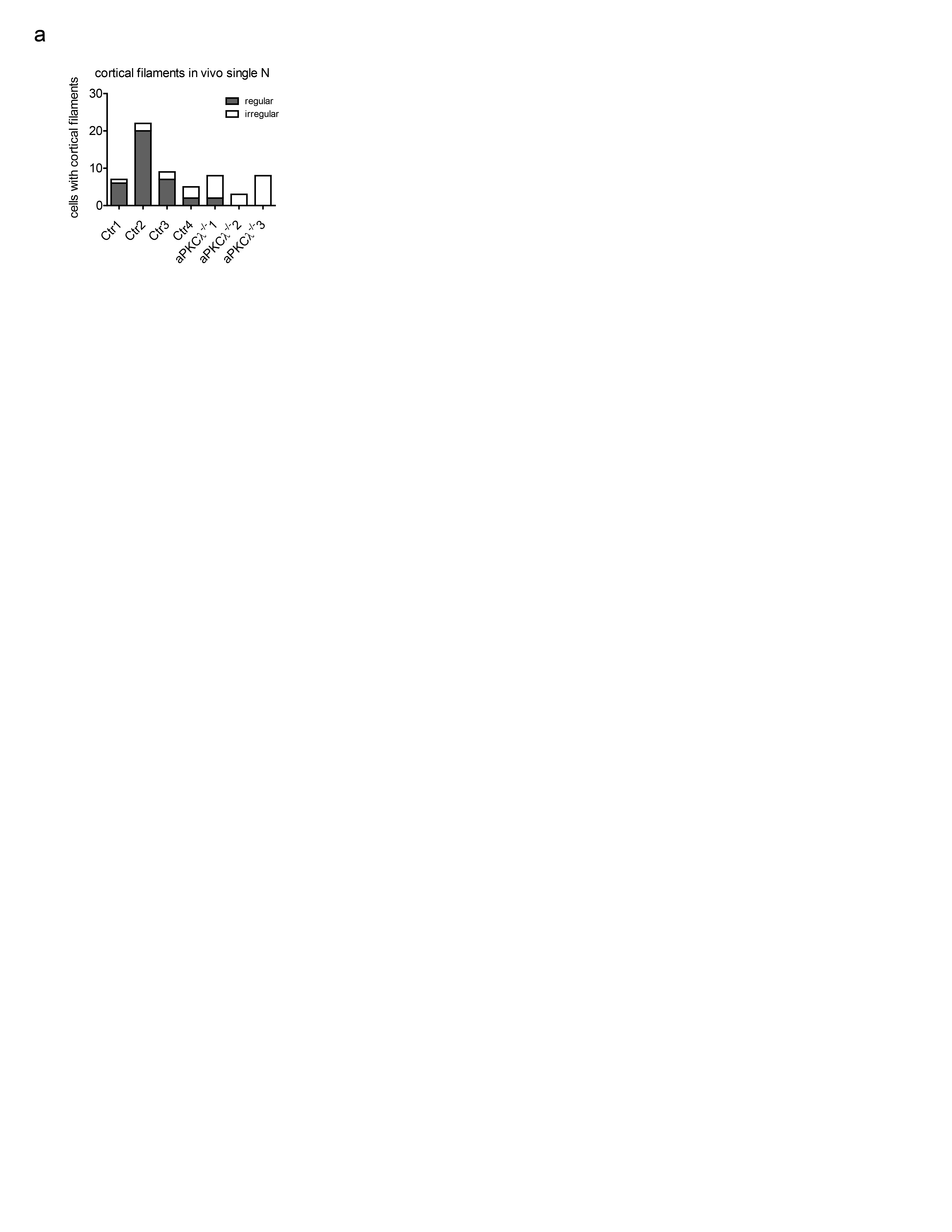


**Fig. 5 supplement. a** Quantification of spinous cells with regular (cross sectioned) and irregular (tangentially cut or absent) filament bundles. Absolut counted cell numbers from each mouse.

**Supplementary video 1.** Keratinocyte sheet rupture test. Live imaging of a stratified keratinocyte sheet mounted in the tissue stretcher while stretching with constant speed until rupture.

**Supplementary Video 2.** Keratin bundles in Ctr keratinocytes. Transmission electron tomography of stratified keratinocyte sheets (48 h Ca^2+^) showing keratin bundles attached to desmosomes in suprabasal layers of Ctr keratinocytes.

**Supplementary Video 3.** Thick keratin bundles in aPKCλ^-/-^ keratinocytes. Transmission electron tomography of stratified keratinocyte sheets (48 h Ca^2+^) showing thick keratin bundles attached to desmosomes in suprabasal layers of aPKCλ^-/-^ keratinocytes.

**Supplementary Video 4.** Keratinocyte sheet contraction. Live imaging of stratified keratinocyte sheets upon dispase treatment showing actomyosin dependent sheet contraction.
